# Abnormal bone micro-architecture and rod-plate configuration in osteopenic adolescent idiopathic scoliosis (AIS)

**DOI:** 10.1186/1748-7161-10-S1-O14

**Published:** 2015-01-19

**Authors:** Ka Yee Cheuk, Tsz Ping Lam, Ivy Jia Jun Zhang, Zhi Wei Wang, Vivian Wing Yin Hung, Bobby Kin Wah Ng, Arthur Fuk Tat Mak, Jack Chun Yiu Cheng

**Affiliations:** 1 Department of Orthopaedics and Traumatology, The Chinese University of Hong Kong, Hong Kong; 2 Joint Scoliosis Research Center of the Chinese University of Hong Kong and Nanjing University China; 3 Department of Mechanical and Automation Engineering, The Chinese University of Hong Kong, Hong Kong

## Objectives

Multiple studies have documented the presence of systemic osteopenia in AIS. Osteopenia was associated with severe curves and was reported to be one of the significant prognostic factors for curve progression in AIS. This study aimed to evaluate bone quality and bone strength parameters including rod-plate configuration and finite element analysis (FEA) with in vivo High-Resolution Peripheral Quantitative Computed Tomography (HR-pQCT) and to investigate their relationship with osteopenia in AIS Vs normal controls.

## Materials and methods

101AIS and 105 controls between 11-14 years old were recruited. Areal bone mineral density (aBMD) of bilateral femoral necks was measured with Dual Energy X-ray Absorptiometry (DXA). Subjects were classified into the osteopenic (Z-score<or=-1) and non-osteopenic (Z-score>-1) group. Bone Morphometry, volumetric bone mineral density (vBMD) and Trabecular Bone Mcro-architecture were measured using HR-pQCT Structural Model Index (SMI) quantifying the trabecular rod/plate configuration (a higher index indicating more rod-like configuration) and FEA in terms of Stiffness, Failure Load and Apparent Modulus were calculated with a standard algorithm.

## Results

In the AIS group, osteopenic subjects showed higher SMI, lower Stiffness, lower Failure Load and lower Apparent Modulus when compared with non-osteopenic subjects (% difference = 15.5%, -24.5%, -23.1% & -20.5% respectively, all with p<0.001). Similar differences in FEA profiles were noted between osteopenic and non-osteopenic subjects in the control group. In contrast, no significant difference in SMI was found between osteopenic and non-osteopenic controls. When all osteopenic subjects were considered, osteopenic AIS subjects had higher SMI when compared with osteopenic controls (% difference = 9.1%, p=0.012).

## Conclusions

This study showed that osteopenia was associated with lower bone strength and a specific pattern of SMI indicating preponderance of rod-like configuration in AIS subjects. Notably the association of higher SMI with osteopenia was seen in AIS but not in normal controls, thus providing strong evidences that osteopenia in AIS was different from osteopenia in non-AIS controls. Further investigations exploring the underlying biochemical and biomechanical mechanisms that bring about these specific endophenotypes are warranted for gaining further understanding of the etiopathogenesis of AIS.

This study was supported by Research Grants Council of the Hong Kong S.A.R., China (Project no: 468809 and 468411)

